# Dyslexia: A Bibliometric and Visualization Analysis

**DOI:** 10.3389/fpubh.2022.915053

**Published:** 2022-06-23

**Authors:** Yanqi Wu, Yanxia Cheng, Xianlin Yang, Wenyan Yu, Yuehua Wan

**Affiliations:** ^1^Institute of Information Resource, Zhejiang University of Technology, Hangzhou, China; ^2^Library, Zhejiang University of Technology, Hangzhou, China

**Keywords:** dyslexia, children, health, bibliometric, keywords analysis

## Abstract

Dyslexia is a disorder characterized by an impaired ability to understand written and printed words or phrases. Epidemiological longitudinal data show that dyslexia is highly prevalent, affecting 10–20% of the population regardless of gender. This study aims to provide a detailed overview of research status and development characteristics of dyslexia from types of articles, years, countries, institutions, journals, authors, author keywords, and highly cited papers. A total of 9,166 publications have been retrieved from the Social Sciences Citation Index (SSCI) and Science Citation Index Expanded (SCI-E) from 2000 to 2021. The United States of America, United Kingdom, and Germany were the top three most productive countries in terms of the number of publications. China, Israel, and Japan led the Asia research on dyslexia. University of Oxford had the most publications and won first place in terms of h-index. *Dyslexia* was the most productive journal in this field and Psychology was the most used subject category. Keywords analysis indicated that “developmental dyslexia,” “phonological awareness,” children and fMRI were still the main research topics. “Literacy,” “rapid automatized naming (RAN),” “assessment,” “intervention,” “meta-analysis,” “Chinese,” “executive function,” “morphological awareness,” “decoding,” “dyscalculia,” “EEG,” “Eye tracking,” “rhythm,” “bilingualism,” and “functional connectivity” might become the new research hotspots.

## Introduction

The term dyslexia is derived from the Greek script and was first proposed in 1887 by Dr. Rudolf Berlin in his work “Eine besondere Art der Wortblindheit (Dyslexie)”. In 1994, Lyon proposed a working definition of dyslexia, and later in 2002, a revised version of dyslexia was approved as “dyslexia is a specific learning that is neurobiological in origin that is characterized by difficulties with accurate and/or fluent word recognition and by poor spelling and decoding abilities” (1, 2). In 1896, Hinshelwood J. published a case of dyslexia (3). There were almost no publications on dyslexia from 1900 to 1945 and the possible reason might be the turmoil of society and the world wars. Since 1946, more scientific research gradually uncovers the reasons behind dyslexia including the causes, symptoms, clinical diagnosis, and improvement measures (4–13). Although the root cause of dyslexia is still unclear, researchers do have some explanations that give us a better understanding of dyslexia and people with dyslexia (14–21). According to the European dyslexia association (EDA), the incidence of dyslexia worldwide is about 9–12%. At present, some countries have passed a series of legislation to promote better identification of people with dyslexia, and to protect the rights in education, employment, and access to public services of individuals with dyslexia (22–25).

Bibliometrics was proposed by Alan Pritchard in 1969, defined as “the application of mathematics and statistical methods to books and other media of communication” (26). Bibliometrics is an important branch of information science and philology. At the same time, it also shows important methodological value and becomes a special research method of information science. The number of bibliometrics academic papers published each year around the world is continually increasing, with about 3,000 in 2021. Bibliometric analyses are useful tools to quantitatively analyze academic literature to get a good understanding of the research trends in specific areas of science and technology, such as public health care (27–32), drug discovery (33–35), nursing (36, 37), biomass (38–42), and COVID-19 (43–49). Bibliometrics has become an academic link closely related to science communication and basic theories. To our knowledge, few comprehensive bibliometric studies have been performed on the dyslexia research literature. Ram (50) conducted an analysis of dyslexia literature (1967–2016) from Scopus, which mainly studied the document types, trends of the number of publications, most productive countries, journals, authors, and keywords. Recently, Zhang et al. (51) published a paper on the top 100 most-cited studies of dyslexia research. Due to the language and the stages of cognition of dyslexia, there is still a need to carry out a comprehensive analysis on the differences of bibliometric characters and research priorities and hotspots of dyslexia research from a country perspective.

To fill this research gap, this study (1) uses the bibliometric method to indicate the status and development trends using major research areas, productive institutes, and journals from a country perspective, (2) analyzes the collaboration patterns between countries and organizations, (3) explores the priorities and hotspots by analyzing the author keywords from temporal evolution and a country perspective. This study demonstrates the status of studies of dyslexia from a country perspective, which offers readers a fresh perspective and suggestions to dyslexia students and families, researchers, and policymakers for future challenges and policy formulation.

## Methods

The analysis was based on the publications related to “dyslexia” which were retrieved through the Social science citation index (SSCI) and science citation index expand (SCI-E) during the period 2000 to 2021. The data were obtained from the Web of Science (WoS) Core Collection by searching the title, abstract, author keywords, and KeyWords plus with search formula of “dyslexia” on January 14th, 2022. The graphical analysis of cooperation uses bibliographic coupling, co-citation, citation, co-authorship, and co-occurrence metrics. We used the Derwent Data Analyzer (DDA) software to present the outcomes of bibliometric analyses. Articles originating from England, Scotland, Wales, and Northern Ireland were grouped under the United Kingdom (UK) heading. The impact factor (IF) for each journal was determined according to the report from the 2020 Journal Citation Reports (JCR). Note that some related publications that did not use “dyslexia” in their topic parts may not be included in this analysis. This issue might produce some deviations.

## Results

### General Statistics

In total, 9,166 papers were obtained from the WoS, including 14 article types. They were articles (7,651), review articles (589), meeting abstracts (409), editorial materials (262), proceedings papers (248), early access (127), letters (101), book reviews (97), corrections (31), book chapters (24), news items (21), biographical-items (4), retracted publications (2), and reprints (1). The vast majority of publications were published in English (8,776; 95.745%), followed by German (197; 2.419%), French (79; 0.862%), Spanish (62; 1.480%), Portuguese (12; 0.131%), Czech (10; 0.109%), and others (30; 0.330%). The following analysis was based on the top eight document types which are the majority of the publications in this field.

**Figure 1:**
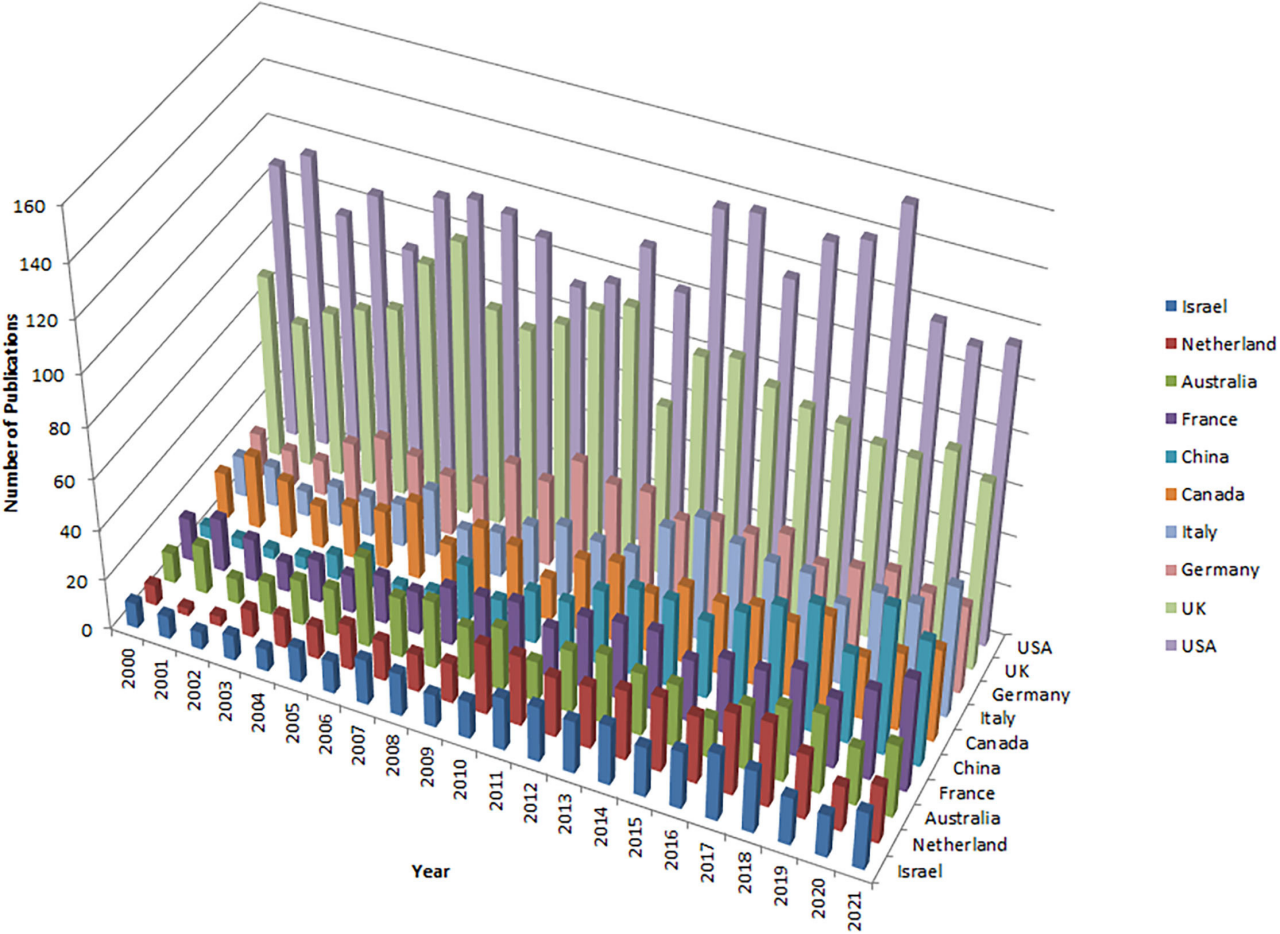
The number of publications per countries by year.

Total 99 countries have published articles on the topic of dyslexia from 2000 to 2021. Figure 1 show the annual analysis of published papers of the top 10 most productive countries. The United States of America published the most articles (2,589) and the highest h-index (148). United Kingdom was in the second position with a total of 1,811 publications. Other productive countries included Germany (721), Italy (648), Canada (598), China (564), France (558), Australia (506), the Netherlands (445), and Israel (380). From 2000 to 2007, the annual output of publications in China did not exceed 10. Thereafter, the number of publications increased rapidly and reached 59 in 2020. In summary, no countries from Africa, and although publications from Asia countries (China and Israel) have increased quickly in the past 10 years, publications from the United States of America and European countries have dominated the dyslexia research field because of their longer accumulation of expertise.

### International Cooperation Analysis

**Figure 2:**
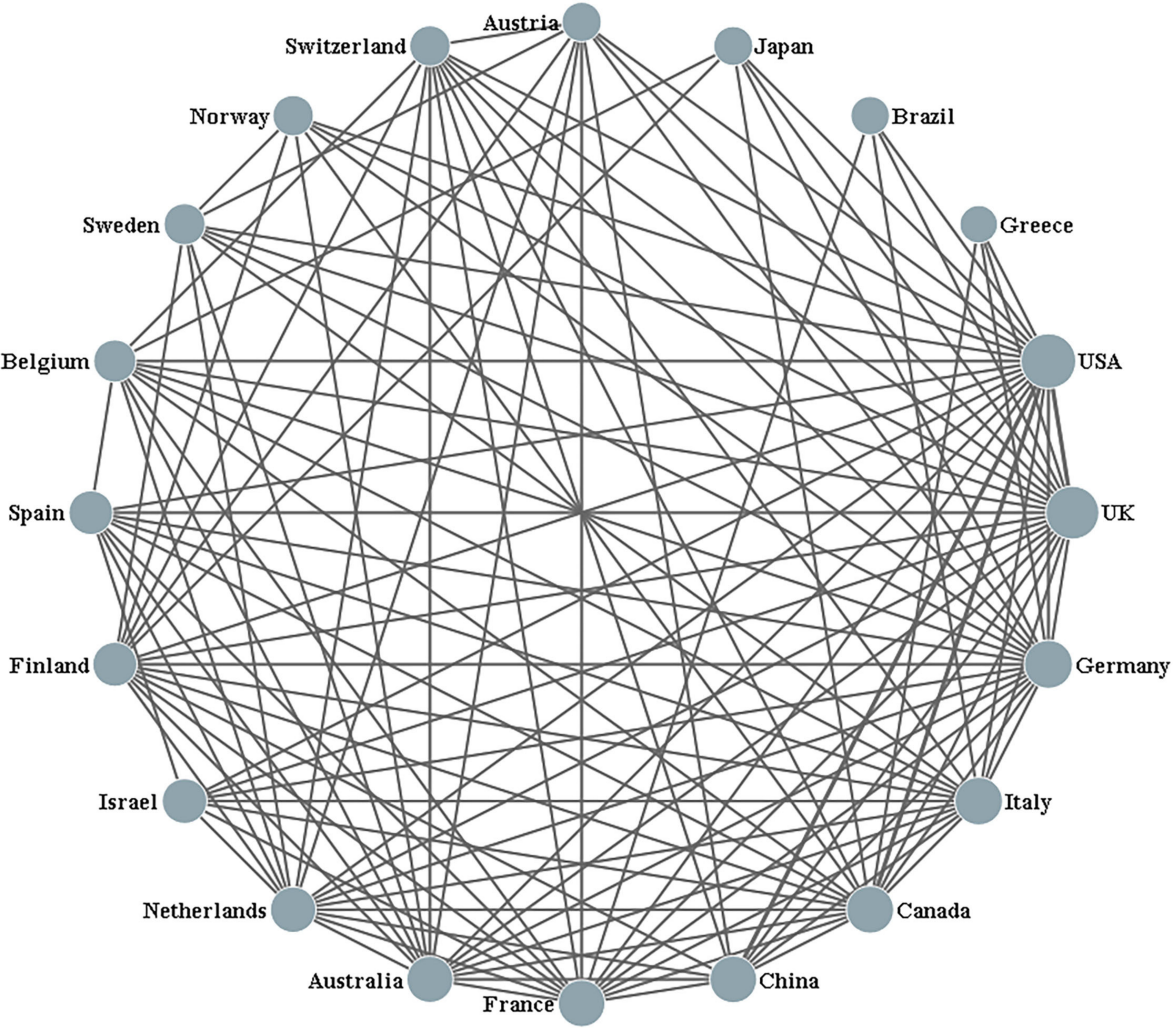
Collaborative relationships among the top 20 most productive countries.

The academic collaboration networks of countries were extracted using Derwent Data Analyzer (DDA) software based on the co-occurrence matrix of author's country and country cooperation. The result of the top 20 most productive countries' cooperation (with a minimum of 5 shared publications) is shown in Figure 2. The size of nodes represents the number of publications. The lines between the nodes represent the cooperative frequency. The United States of America is the country with the highest number of papers in the dyslexia research field, followed by the United Kingdom, Germany, Italy, Canada, and China. As can be seen in Figure 2, the United States of America cooperated most frequently with the United Kingdom, Canada, and China. Furthermore, the United States of America and United Kingdom had the biggest collaboration network among the top 20 most productive countries. Researchers from Japan, Brazil, and Greece need to strengthen their international cooperation. China, Israel, and Japan led the research in Asian countries.

### Organization Co-occurrence Analysis

**Figure 3:**
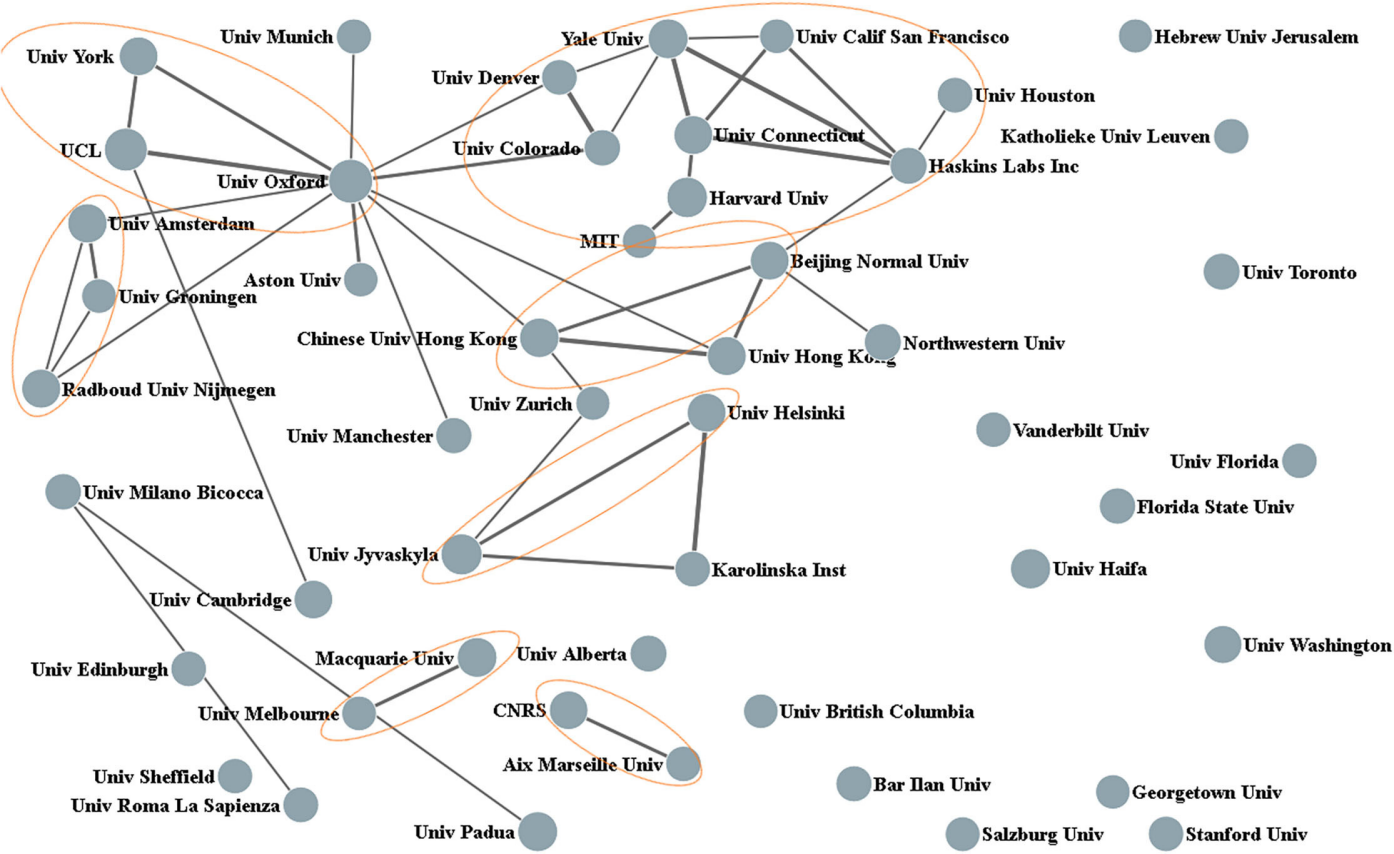
Collaborative relationships among the top 50 most productive organizations.

A total of 4,869 organizations have published papers on the study of dyslexia. The top 15 most productive organizations concerning the number of publications and h-index have been enlisted in Table 1. The University of Oxford ranked first in terms of total publications and obtained the highest h-index (75), followed by UCL and the University of Jyvaskyla. Yale University has the highest number of ACCP. Figure 3 shows the cooperation between organizations with a minimum of 8 papers among the top 50 productive organizations. As shown in Figure 3, institutions from the same country were more closely connected. This was confirmed by the analysis of the top 3 most collaborative organizations for each institution (see Table 1). The University of Oxford has the largest collaborative network.

**Table 1:** The top 15 most productive organizations of publication, citations and h-indices during 2000–2021.

**Organizations**	**TP**	**ACCP**	**h-index**	**SP (%)**	**Country**	**Top 3 most collaborative organization**
Univ Oxford	318	55.09	75	75.16	UK	UCL, Univ York, Aston Univ
UCL	266	54.02	61	100.00	UK	Univ Oxford, Univ York, Univ London
Univ Jyvaskyla	192	46.60	53	70.83	Finland	Univ Helsinki, Niilo Maki Inst, Karolinska Inst
Harvard Univ	176	48.68	47	93.75	USA	Beth Israel Deaconess Med Ctr, Univ Connecticut, Massachusetts Gen Hosp
Univ Haifa	163	25.07	31	50.92	Israel	Northwestern Univ, Ankara Univ, Carnegie Mellon Univ
Univ Padua	156	47.31	42	86.54	Italy	Univ Bergamo, Sci Inst E Medea, CNR
Yale Univ	154	58.70	46	90.26	USA	Haskins Labs Inc, Univ Connecticut, Moscow MV Lomonosov State Univ
Macquarie Univ	152	41.12	33	73.03	Australia	Univ Melbourne, Univ Alberta, Childrens Hosp Westmead
Chinese Univ Hong Kong	144	38.68	41	83.33	P. R. China	Univ Hong Kong, EDUHK, Beijing Normal Univ
Univ Amsterdam	141	33.89	33	70.92	Netherland	Univ Groningen, Iwal Inst, Rudolf Berlin CTR
Radboud Univ Nijmegen	133	23.22	30	74.44	Netherland	Max Planck Soc, Univ Groningen, Univ Oxford
Univ Hong Kong	132	30.33	31	78.79	P. R. China	Chinese Univ Hong Kong, EDUHK, Beijing Normal Univ
Univ Connecticut	130	34.63	33	90.00	USA	Yale Univ, Haskins Lab, Harvard Univ
Univ Helsinki	129	41.60	40	89.15	Finland	Helsinki Univ Hosp, Karolinska Inst, Univ Jyvaskyla
Beijing Normal Univ	128	31.91	33	95.31	P. R. China	Chinese Univ Hong Kong, Peking Univ, Chinese Acad Sci

*TP, total paper; ACCP, average citations per paper; SP, Share of publications*.

In addition, we analyzed the share of cooperative publications between institutes (see Table 1). It can be seen that all the 15 most productive institutions except University Haifa have very high collaboration rates, especially the UCL, Harvard University, Yale University, and the University of Connecticut. Interestingly, all of the top 15 prolific organizations are universities. It suggests that dyslexia research is mostly held by universities.

### Prolific Authors' Analysis From a Country Perspective

There are 17,009 authors who have published at least one paper on the research of dyslexia. Table 2 outlines the top 20 contributing authors based on the number of publications they authored or co-authored. As can be seen in these data, all of the authors are from the top 20 productive countries. Among the top 20 prolific authors, five authors are from the united States of America, four from United Kingdom, two from Finland, P. R. China, Belgium, and Italy, and one from Germany, the Netherlands, and Austria. Schulte-korne G. ranked first on the list with the highest number (101) of dyslexia papers, Snowling MJ obtained the second one with 99 papers, and Lyytinen H obtained the third one with 95. For the average citation per paper, Fletcher JM ranked first with 99.63, followed by Pennington BF (86.49) and Bishop DVM (81.98). Looking to the H-index record, Lyytinen H obtained the first position with 45, followed by Snowling MJ, Goswami U, and Pennington BF. It is worth noting that three out of four United Kingdom researchers are from the same institution, the University of Oxford. Once again it proved the outstanding contribution of the University of Oxford to dyslexia research.

**Table 2:** The top 20 most productive authors of publication, and h-indices during 2000–2021.

**Rank**	**Author**	**TP**	**TC**	**TC/TP**	**h-index**	**Affiliation (latest address)**
1	Schulte-korne G	101	2,748	27.21	30	Univ Hosp Munich, Dept Child and Adolescent Psychiat and Psychotherapy, Munich, **Germany**
2	Snowling MJ	99	8,040	81.21	43	Univ Oxford, Dept Expt Psychol, Oxford, **UK**
3	Lyytinen H	95	6,790	71.47	45	Univ Jyvaskyla, Niilo Maki Inst, **Finland**
4	Goswami U	89	6,803	76.44	42	Univ Cambridge, Ctr Neurosci Educ, **UK**
5	Pennington BF	73	6,314	86.49	39	Univ Denver, Dept Psychol, Denver, **USA**
6	Berninger VW	68	3,287	48.34	35	Univ Washington, Dept Educ Psychol, **USA**
7	Ghesquiere P	67	2,253	33.63	25	Fac Psychol & Educ Sci, Leuven, **Belgium**
7	Hulme C	67	3,901	58.22	33	Univ Oxford, Dept Educ, Oxford, **UK**
9	Zoccolotti P	65	1,829	28.14	24	IRCCS Fdn Santa Lucia, Dev Dyslexia Lab; Sapienza Univ Rome; **Italy**
10	Shu H	61	2,953	48.41	29	Beijing Normal Univ, State Key Lab Cognit Neurosci & Learning, **P. R. China**
10	Olson RK	61	4,115	67.46	34	Univ Nebraska Med Ctr, Dept Neurol Sci, **USA**
12	Leppanen PHT	60	2,542	42.37	27	Univ Jyvaskyla, Dept Psychol, Jyvaskyla, **Finland**
13	Fletcher JM	60	5,978	99.63	30	Univ Houston, Houston, **USA**
14	Bishop DVM	58	4,755	81.98	35	Univ Oxford, Dept Expt Psychol, Oxford, **UK**
15	Facoetti A	56	3,330	59.46	31	Univ Padua, Dept Gen Psychol, Dev & Cognit Neurosci Lab, **Italy**
15	Monaco AP	56	3,748	66.93	33	Tufts Univ, Medford, **USA**
17	Landerl K	54	2,523	46.72	21	Karl Franzens Univ Graz, Inst Psychol, Univ Pl 2, **Austria**
18	Ho CSH	53	1,943	36.66	24	Univ Hong Kong, Dept Psychol, Hong Kong, **Peoples R China**
19	Wouters J	53	2,032	38.34	23	Katholieke Univ Leuven, Dept Neurosci, Res Grp ExpORL, **Belgium**
20	Verhoeven L	52	673	12.94	16	Radboud Univ Nijmegen, Behav Sci Inst, Nijmegen, **Netherlands**

*TA, total publications; TC, total citations*.

Schulte-korne G. is from Ludwig-Maximilians-University of Munich and his main research areas in dyslexia include genetics (52–56), assessment (57, 58), intervention (59–62), language (63, 64), and cognitive neuroscience (55, 65, 66). Snowling MJ, listed in the second place, is from the University of Oxford and her research on dyslexia focuses on language impairment (67–69), comorbidity (70, 71), and intervention (72–76). Lyytinen H is from the University of Jyvaskyla and his research on dyslexia focuses on the longitudinal study (21, 77–79), speech perception (80–82), auditory processing (83–85), and intervention (86, 87).

### Research Area and Journal Analysis From a Country Perspective

**Figure 4:**
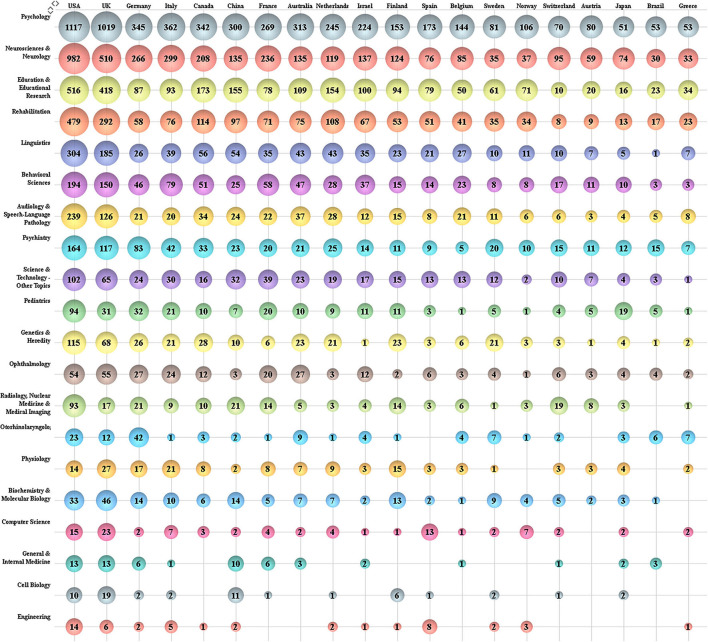
Number of papers in the top 20 research areas by the top 20 most productive countries.

Research works on dyslexia have been carried out in about 101 research areas in SCI and SSCI databases. Figure 4 shows the number of papers published by the top 20 most productive countries in the top 20 most productive research areas. “Psychology” ranked first in terms of the total publications of all countries. “Neurosciences Neurology” and “Education Educational Research” are listed in the second or third position in all countries. Sweden, Spin, Norway, the Netherlands, China, and Greece had published more papers on “Education Educational Research” than “Neurosciences Neurology”.

The 9,110 papers related to dyslexia research during 2000–2021 were published in 1,156 journals. Table 3 shows the number of papers published by the top 15 most productive countries in the top 10 most productive journals. About 30% of articles were published in these top 10 productive journals in the top 15 countries. *Dyslexia* published the most articles in this research field (415 publications), followed by *Neuropsychologia* (302), *Journal of Learning Disabilities* (296), and *Frontiers in Psychology* (280). United Kingdom published the most articles in *Dyslexia* while United States of America published more articles in the *Journal of Learning Disabilities* and *Annals of Dyslexia* than other countries. These suggested that the United Kingdom and United States of America researchers prefer to publish in journals hosted by their countries.

**Table 3:** Number of papers in the top 10 Journals and by the top 15 most productive countries.

	**Dyslexia**	**Journal of learning disabilities**	**Neuropsychologia**	**Frontiers in psychology**	**Annals of dyslexia**	**Reading and writing**	**Brain and language**	**Scientific studies of reading**	**Cortex**	**PLoS ONE**
USA	42	116	65	47	81	57	74	46	39	40
UK	124	19	56	36	17	33	36	29	46	21
Germany	14	7	28	23	12	11	10	11	6	9
Italy	23	18	36	37	9	8	16	4	20	11
Canada	28	40	20	9	22	25	20	19	6	7
China	20	15	12	35	20	31	11	11	4	16
France	16	7	25	16	13	2	9	5	17	21
Australia	26	8	15	5	6	12	4	12	13	6
Netherland	36	15	11	11	21	16	8	22	8	9
Israel	17	12	12	14	18	15	4	6	15	12
Finland	12	21	3	7	9	13	5	8	2	4
Spain	11	10	5	15	17	8	3	8	4	2
Belgium	6	2	8	16	6	6	11	4	10	8
Sweden	15	5	1	1	8	8	3	6	1	8
Norway	25	3	5	8	2	11	2	8	1	1

### An Analysis of Author Keywords From a Global Perspective

Keywords analysis has been used widely to analyze research hotspots and trends (88–93). To identify the research focus of dyslexia research, 9,562 author keywords which appeared 32,757 times from 9,110 papers were analyzed. Keywords with the same meanings were grouped and represented by one unified word or phrase, and the publications that lack author keywords may not be included in this analysis. Among the author keywords, 6,705 (70%) were used only once. The high percentage of once-only author keywords may indicate a lack of continuity in research and a wide range of interests in dyslexia research.

**Figure 5:**
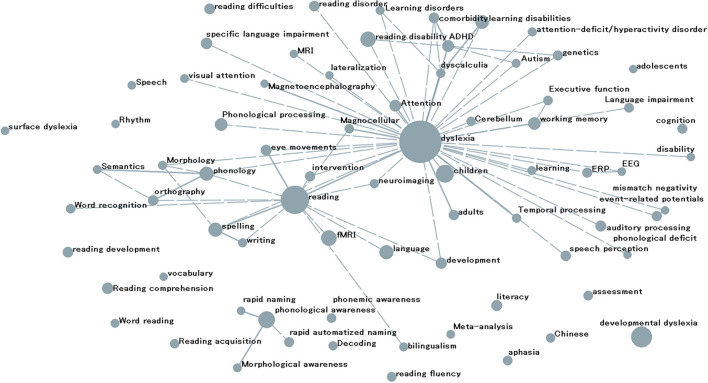
Globe research hot points related to dyslexia.

Figure 5 shows a network map of author keywords co-occurrence analysis (frequency not <50 times) related to dyslexia. As seen in the analysis result in Figure 5, the keywords “dyslexia” and “reading” occupied the core positions. The top high-frequency nodes linked with “dyslexia” are “reading,” “children,” “attention,” “dyscalculia,” “magnocellular,” “adults,” “magnetoencephalography,” and “MRI”. Keywords “fMRI,” “eye movements,” “spelling,” “intervention,” “phonology,” and “writing” were the top high-frequency nodes connected to “reading”.

To better understand the development trend of research, we compared the top 50 high-frequency author keywords in the past 5 years and the first 16 years (see Table 4); “dyslexia” and “developmental dyslexia” were exceptions because these keywords were among the search terms of the data that were used in this study. “Phonological awareness,” “reading,” and “spelling” are the main research aspects; “children” are the main group studied. “fMRI” was still a strong and useful technique to measure the brain activity of dyslexia and remained among the top eight most frequently used keywords (94, 95). “Literacy” refers to the quality or state of being literate, especially the ability to read and write. The rank of “literacy” increased from 27th in 2000–2016 to 15th in 2017–2021, suggesting that the research of literacy remained hot research during the past 20 years.

**Table 4:** Temporal evolution of the 50 most frequency used author keywords.

**2000–2016**		**2017–2021**	
**Rank**	**Author keywords**	**Rank**	**Author keywords**
1	Dyslexia	1	Dyslexia
2	Reading	2	Reading
3	Developmental dyslexia	3	Developmental dyslexia
4	phonological awareness	4	Phonological awareness
5	Children	5	Children
6	Reading disability	6	fMRI
7	Attention deficit hyperactivity disorder (ADHD)	7	Spelling
8	fMRI	8	Reading disability
9	Phonology	9	Attention deficit hyperactivity disorder (ADHD)
10	Language	10	Learning disabilities
11	Spelling	11	Reading difficulties
12	Learning disabilities	12	Reading comprehension
13	Phonological processing	13	Working memory
14	Working memory	14	Language
15	Attention	15	Literacy
16	Specific language impairment	16	Phonology
17	Auditory processing	17	Reading disorder
18	Reading disorder	18	Executive function
19	Development	19	Development
20	Language impairment	20	Eye movements
21	Comorbidity	21	Intervention
22	Event-related potentials	22	Reading development
23	Orthography	23	Comorbidity
24	Speech perception	24	Cognition
25	Eye movements	25	Phonological processing
26	Adults	26	Morphological awareness
27	Literacy	27	Rapid automatized naming
28	Reading development	28	Reading fluency
29	Genetics	29	Assessment
30	ERP	30	Meta-analysis
31	Reading comprehension	31	Decoding
32	Temporal processing	32	Chinese
33	Magnocellular	33	Dyscalculia
34	Aphasia	34	Neurodevelopmental disorders
35	Cerebellum	35	Specific learning disorder
36	Intervention	36	EEG
37	Rapid automatized naming	37	Eye tracking
38	Cognition	38	Phonemic awareness
39	Word recognition	39	Reading acquisition
40	Assessment	40	Attention
41	Learning disorders	41	Developmental language disorder
42	Phonemic awareness	42	Rhythm
43	Reading acquisition	43	Adults
44	Semantics	44	Bilingualism
45	Reading difficulties	45	Disability
46	Reading fluency	46	Morphology
47	Visual attention	47	Neuroimaging
48	Autism	48	Aphasia
49	Chinese	49	Functional connectivity
50	Lateralization	50	Word recognition

With the in-depth research and experience accumulation of dyslexia, early intervention and prevention of dyslexia have important social significance (96). “Rapid automatized naming (RAN)” as one of the effective cognitive measures drew the attention of researchers that involved in creating optimal assessments and interventions (97). In fact, the rank of “Rapid automatized naming” had an apparent upward movement from 37th in 2000–2016 to 27th in 2017–2021. Both “Assessment” and “intervention” also had a big upward movement from 40th in 2000–2016 to 29th in 2017–2021 and 36th in 2000–2016 to 21st in 2017–2021, respectively. In 1976, Gene Glass first used the name “Meta-analysis” to represent the process and method of integrating and analyzing many empirical studies on the same subject through statistical analysis to obtain the most representative conclusions. This method had become an important tool for analyzing various research results of dyslexia (98–101) and “meta-analysis” reached the 30th in 2017–2021 from 95th in 2000–2016.

In the 1980s and 1990s, Chinese scholars began to study dyslexia in reading Chinese, but most of the results were published in their Chinese journals. In recent years, with the enhancement of scientific research capabilities and international cooperation, increased research results have been published in international journals (102–106). The rank of “Chinese” had a dramatic increase from 49th in 2000–2016 to 32nd in 2017–2021. It is also worth mentioning that “executive function” (107, 108), “morphological awareness” (109, 110), “Meta-analysis,” “decoding” (16), “dyscalculia,” “EEG” (111, 112), “Eye tracking” (113, 114), “rhythm” (115, 116), “bilingualism” (117, 118), and “functional connectivity” (119, 120) entered the top 50 high-frequency keywords in 2017–2021, suggesting that these topics may become the new research hotspots.

### An Analysis of Author Keywords From a Country Perspective

Table 5 shows the 20 countries with the highest scientific production in dyslexia research as well as the keywords most used by these countries. Not surprisingly, “dyslexia,” “reading,” and “developmental dyslexia” were the keywords most used by most of these countries, ranking first to third in 15 of the 20 countries. “fMRI” was one of the research hotspots in the United States of America, Norway, Switzerland, and Austria. The language of early research on dyslexia was mainly English. In the 1970s, some researchers believed that the writing system of Asian countries would not cause dyslexia. However, with the development of early reading education activities by educators in some Asian countries, the problem of children's dyslexia had gradually attracted the attention of researchers. Therefore, it was not surprising that Asian countries (China, Japan, and Israel) had their language as one of their research focuses (104, 121–128).

**Table 5:** Top 5 most used author keywords by top 20 most productive countries.

**Country**	**Top 5 highly used author keywords**
USA	Dyslexia, reading, reading disability, fMRI, language
UK	Dyslexia, reading, developmental dyslexia, phonology, language
Germany	Dyslexia, reading, children, developmental dyslexia, phonological awareness
Italy	Dyslexia, reading, developmental dyslexia, children, neglect dyslexia, working memory
Canada	Dyslexia, reading, reading disability, developmental dyslexia, phonological awareness
China	Dyslexia, developmental dyslexia, Chinese, reading, children
France	Dyslexia, developmental dyslexia, reading, children, Visual attention span
Australia	Dyslexia, reading, phonological awareness, children, magnocellular, spelling
Netherlands	Dyslexia, reading, developmental dyslexia, phonological awareness, reading development
Israel	Dyslexia, reading, developmental dyslexia, Hebrew, phonological awareness
Finland	Dyslexia, reading, developmental dyslexia, mismatch negativity, reading difficulties
Spain	Dyslexia, reading, developmental dyslexia, Spanish, ADHD
Belgium	Dyslexia, reading, developmental dyslexia, speech perception, Phonological processing
Sweden	Dyslexia, reading, phonological awareness, ADHD, developmental dyslexia
Norway	Dyslexia, reading, reading difficulties, fMRI, phonological awareness
Switzerland	Dyslexia, reading, developmental dyslexia, children, fMRI
Austria	Dyslexia, reading, developmental dyslexia, fMRI, spelling
Japan	Dyslexia, reading, developmental dyslexia, Japanese, phonological awareness
Brazil	Dyslexia, reading, Phonological processing, children, phonemic awareness
Greece	Dyslexia, reading, Magnetoencephalography, functional brain imaging, phonological decoding

### An Analysis of Highly Cited Papers Based on WoS

The citation account is an important indicator of academic influence and was widely used in research evaluation. According to the Essential Science Indicators (ESI) database, highly cited papers (HCPs) refers to papers with citations in the top 1% of all papers based on a cited threshold for an academic field and publication year during the past 10 years. To some extent, HCPs from the ESI database might reflect research directions and hotspots in an academic field (129). Table 6 shows the HCPs of dyslexia over the last 10 years. One was published in the *Lancet* (IF = 79.323 in 2020) and *Nature Reviews Neuroscience* (IF = 34.87 in 2020). Two were published in the *Annual Review of Psychology, Journal of Learning Disabilities*, and *Trends in Cognitive Sciences*, respectively. Among the 16 HCPs, seven papers included authors from the United States of America and the United Kingdom, two from Germany, Finland, and Norway, and one from Finland, China, Austria, France, Hungary, Switzerland, and the Netherlands. It is worth mentioning that China was the only non-European and non-United States country, indicating that China has strengthened its development in this field of research. Among the 16 HCPs, two were about Rapid Automatized Naming (RAN) (97, 130) and two about meta-analysis (100, 101), indicating that RAN and meta-analysis became the hotspots in dyslexia research. “Predictors of developmental dyslexia” (131) and “Early detection of dyslexia risk” (96) might be one of the new dyslexia research directions.

**Table 6:** Highly-cited papers of dyslexia.

**Authors**	**Title**	**Journal**	**Country**	**Year**
Fletcher, JM; Francis, DJ; Foorman, BR; et al.	Early detection of dyslexia risk: development of brief, teacher-administered screens	Learning Disability Quarterly	USA	2021
Ullman, MT; Earle, FS; Walenski, M; et al.	The neurocognition of developmental disorders of language	Annual Review of Psychology, Vol 71	USA	2020
Stein, J	The current status of the magnocellular theory of developmental dyslexia	Neuropsychologia	UK	2019
Landerl, K; Freudenthaler, HH; Heene, M; et al.	Phonological awareness and rapid automatized naming as longitudinal predictors of reading in five alphabetic orthographies with varying degrees of consistency	Scientific Studies of Reading	Germany	2019
Snowling, MJ; Melby-Lervag, M	Oral language deficits in familial dyslexia: a meta-analysis and review	Psychological Bulletin	UK/Norway	2016
Goswami, U	Sensory theories of developmental dyslexia: three challenges for research	Nature Reviews Neuroscience	UK	2015
Peterson, RL; Pennington, BF	Developmental dyslexia	Annual Review of Clinical Psychology, Vol 11	USA	2015
Willcutt, EG; Petrill, SA; Wu, S; et al.	Comorbidity between reading disability and math disability: concurrent psychopathology, functional impairment, and neuropsychological functioning	Journal of Learning Disabilities	USA	2013
Hamalainen, JA; Salminen, HK; Leppanen, PHT	Basic auditory processing deficits in dyslexia: systematic review of the behavioral and event-related potential/field evidence	Journal of Learning Disabilities	Finland	2013
Landerl, K; Ramus, F; Moll, K; et al.	Predictors of developmental dyslexia in European orthographies with varying complexity	Journal of Child Psychology and Psychiatry	Austria/France/UK/Finland/ Germany/Hungary/ Switzerland/Netherland/ USA	2013
Li, H; Shu, H; McBride-Chang, C; et al.	Chinese children's character recognition: Visuo-orthographic, phonological processing and morphological skills	Journal of Research in Reading	China	2012
Peterson, RL; Pennington, BF	Developmental dyslexia	Lancet	USA	2012
Melby-Lervag, M; et al.	Phonological skills and their role in learning to read: a meta-analytic review	Psychological Bulletin	UK/ Norway	2012
Norton, ES; Wolf, M	Rapid automatized naming (RAN) and reading fluency: implications for understanding and treatment of reading disabilities	Annual Review of Psychology, Vol 63	USA	2012
Price, CJ; Devlin, JT	The Interactive Account of ventral occipitotemporal contributions to reading	Trends in Cognitive Sciences	UK	2011
Goswami, U	A temporal sampling framework for developmental dyslexia	Trends in Cognitive Sciences	UK	2011

*As of November/December 2021, these highly cited papers received enough citations to place it in the top 1% of the academic field of Social Sciences, general based on a highly cited threshold for the field and publication year. Data collection: 2022-05-12*.

## Discussion

There is no doubt that more countries have taken dyslexia seriously over the past few decades. The United States of America, United Kingdom, and Germany had done well in publishing research papers in this field. Some Asian countries like China and Israel have started to play a role in dyslexia research. It is worth noting that in 2020–2021, the research results from China increased significantly, and the ranking jumped to third place based on the number of published papers in the past 2 years.

North America, Western and Northern Europe, Asia, and Australia were the most active regions in the research of dyslexia. This was further confirmed by most active institutions and authors. There were no organizations from Africa in the top 15 most productive institutions that indicated that the issues relating to dyslexia in low-income regions lag far behind in developed countries and regions. The possible reason might be poor awareness of dyslexia among educators, the public, funding input, economic level, etc. As dyslexia is a world health issue, we expect more Asian and African nations join this research area. Although, most of the dyslexia research is held by universities, it will benefit sharing its knowledge and experiences between organizations such as hospitals, schools, and research centers.

According to the keywords analysis, 65% of publications were about children, suggesting that the most of research was about children with dyslexia. At present, MRI technology is mostly used to explore the brain function and mechanism of dyslexia, among which fMRI research is at the forefront. As can be seen from Figure 5 and Tables 4, 6, “developmental dyslexia,” “phonological awareness,” children, and fMRI are still the hotspots in dyslexia research. By comparing the keywords in papers published before and after 2017, we found that the keywords “literacy,” “rapid automatized naming (RAN),” “assessment,” “intervention,” “meta-analysis,” “Chinese,” “executive function,” “morphological awareness,” “decoding,” “dyscalculia,” “EEG,” “Eye tracking,” “rhythm,” “bilingualism,” and “functional connectivity” were increasingly attracting the attention of researchers and had become some new research hotspots in dyslexia research. With the rapid development of the Internet, more knowledge is mainly obtained through network resources, and the effect of dyslexia on “information seeking” behavior has gradually attracted the attention of dyslexia researchers (132, 133). In addition, the emergence of a new keyword COVID-19 in the past 2 years also showed that during the COVID-19 epidemic, researchers began to study the impact of the epidemic on dyslexia research (115, 134–137). As the international exchange of dyslexia research continues to grow, scientists are aware that differences in education-related legislation in different countries may lead to persistent differences between psychologists' assessment practices. “Methods used by psychologists for identifying dyslexia: A systematic review” by Sadusky et al. (138) drew a conclusion that “a consensus operational definition of dyslexia and universal assessment guidelines” is needed. At the same time, the public library, as one of the important places for people to read, has thought about how to better serve dyslexic users (139, 140).

## Conclusions

In this study, we presented a general overview of the dyslexia research area from a country perspective. The number of countries participating in dyslexia research increased to 68 in 2021 from 32 in 2000. In total, 99 countries published papers in this research field since 2000. All 9,110 publications were analyzed based on co-occurrence of country, institution, author, and author keyword. The United States of America, United Kingdom, and Germany were the top three most prolific countries and had the biggest collaboration network in the dyslexia research. Currently, international cooperation is still insufficient in Asian and African countries. The advanced expertise and experience of developed countries can be shared with developing countries through international cooperation. To our knowledge, there is no cure for dyslexia, but early assessment and intervention will give the best outcome. And also, people with dyslexia can learn to read with structured literacy which helps to rewire their brains. This was confirmed by the topmost used author keywords “intervention,” “assessment,” and “literacy”.

This study provided an insight into the status of current dyslexia research. It can also provide useful information for relevant researchers to find potential collaborators. In addition, this study may help to increase public awareness and acceptance of dyslexia, disseminate knowledge of dyslexia to educators, policymakers, and especially parents of children with dyslexia.

## Data Availability Statement

The original contributions presented in the study are included in the article/supplementary material, further inquiries can be directed to the corresponding author/s.

## Author Contributions

YWan and YC designed the study. WY is responsible for data collection. YC and XY analyzed the data. YWu analyzed, interpreted the data, and wrote the manuscript. All authors have read and agreed to the published version of the manuscript.

## Funding

This research was funded by National Social Science Foundation of China (20BTQ028), the Scientific Research Program of Zhejiang Educational Committee (Y202147067), and Humanities and Social Sciences Research Foundation of Zhejiang University of Technology (SKY-ZX-20200076).

## Conflict of Interest

The authors declare that the research was conducted in the absence of any commercial or financial relationships that could be construed as a potential conflict of interest.

## Publisher's Note

All claims expressed in this article are solely those of the authors and do not necessarily represent those of their affiliated organizations, or those of the publisher, the editors and the reviewers. Any product that may be evaluated in this article, or claim that may be made by its manufacturer, is not guaranteed or endorsed by the publisher.
